# Common genetic variants in pituitary–thyroid axis genes and the risk of differentiated thyroid cancer

**DOI:** 10.1530/EC-12-0017

**Published:** 2012-10-10

**Authors:** Susana Pastor, Abdelmounaim Akdi, Eddy R González, Juan Castell, Josefina Biarnés, Ricard Marcos, Antonia Velázquez

**Affiliations:** 1 Grup de Mutagènesi, Departament de Genètica i de Microbiologia, Facultat de Biociències Universitat Autònoma de Barcelona 08193, Cerdanyola del Vallès Barcelona Spain; 2 CIBER Epidemiología y Salud Pública, ISCIII Barcelona Spain; 3 Servei de Medicina Nuclear Hospital Vall d'Hebron Barcelona Spain; 4 Unidad de Endocrinología Hospital Josep Trueta Girona Spain

**Keywords:** thyroid-function genes, association, thyroid cancer risk, cancer susceptibility

## Abstract

Thyroid hormone receptors, THRA and THRB, together with the TSH receptor, TSHR, are key regulators of thyroid function. Alterations in the genes of these receptors (*THRA*, *THRB* and *TSHR*) have been related to thyroid diseases, including thyroid cancer. Moreover, there is evidence suggesting that predisposition to differentiated thyroid cancer (DTC) is related to common genetic variants with low penetrance that interact with each other and with environmental factors. In this study, we investigated the association of single nucleotide polymorphisms (SNPs) in the *THRA* (one SNP), *THRB* (three SNPs) and *TSHR* (two SNPs) genes with DTC risk. A case–control association study was conducted with 398 patients with sporadic DTC and 479 healthy controls from a Spanish population. Among the polymorphisms studied, only *THRA*-rs939348 was found to be associated with an increased risk of DTC (recessive model, odds ratio=1.80, 95% confidence interval=1.03–3.14, *P*=0.037). Gene–gene interaction analysis using the genotype data of this study together with our previous genotype data on *TG* and *TRHR* indicated a combined effect of the pairwises: *THRB*-*TG* (*P*_interaction_=0.014, *THRB*-rs3752874 with *TG*-rs2076740; *P*_interaction_=0.099, *THRB*-rs844107 with *TG*-rs2076740) and *THRB*-*TRHR* (*P*_interaction_=0.0024, *THRB*-rs3752874 with *TRHR*-rs4129682) for DTC risk in a Spanish population. Our results confirm that *THRA* is a risk factor for DTC, and we show for the first time the combined effect of *THRB* and *TG* or *TRHR* on DTC susceptibility, supporting the importance of gene–gene interaction in thyroid cancer risk.

## Introduction

Thyroid cancer is of special concern in endocrinology practice, accounting for more than 90% of all endocrine cancers. Moreover, its incidence is increasing in developed countries (1, 2). Differentiated thyroid carcinoma (DTC) represents more than 90% of thyroid neoplasms, and it comprises papillary (PTC) and follicular (FTC) histological subtypes (3). The genetic predisposition to thyroid cancer is estimated to be higher than in other common cancers and, together with environmental factors, is considered the main component in the aetiology of thyroid cancer (4, 5, 6, 7).

Association studies have identified several genetic variants related to thyroid cancer risk, including 8q24, 1p12, 9q22.23, 14p13.3 and 5q33 (8, 9, 10, 11, 12). Polymorphic variations in the *FOXE1* and *NKX2-1* genes, both involved in thyroid function and development of the thyroid gland, have also been reported to be associated with the risk of thyroid cancer (10, 13, 14). Furthermore, common genetic variants associated with low levels of TSH and increased thyroid cancer risk suggest the role of thyroid-related hormones in thyroid cancer susceptibility (15), including thyroid hormone receptors (TRs) and TSH receptor (TSHR), which are key proteins in the regulation of the thyroid function. Although mutations in the TR genes, *THRA* and *THRB*, are not common in thyroid tumours (16, 17, 18), a decreased expression of these genes has been found in thyroid cancer (19).

These observations together with a study using mouse models with deleted *Thra* and *Thrb* genes support the tumour suppressor functions of the TR genes (20). Three studies have reported the association of genetic variants in the *THRA* gene with thyroid cancer risk, but the conclusions are limited by the small size of the populations studied (21, 22, 23). In the case of *THRB*, there is no reported data about *THRB* genetic variants related to the thyroid cancer risk. The implication of *TSHR* in thyroid cancer is unclear (24, 25). While epigenetic silencing of *TSHR* in thyroid carcinomas has been reported (26), two other studies have shown a lack of association of *TSHR* polymorphisms with thyroid cancer risk (27, 28). On the contrary, *TSHR* is associated with other thyroid diseases (29).

Given the limited information concerning the implication of genes involved in the thyroid function on thyroid cancer risk, in this study, we have analysed the risk association between common genetic variants in the *THRA*, *THRB* and *TSHR* genes and DTC in a large Spanish population. In addition, we have examined the combined effect of the variants of these three genes and the *TG* and *TRHR* genes on DTC susceptibility, using data from our previous association studies (12).

## Materials and methods

### Subjects

The study was carried out on a Spanish population with a total of 877 unrelated subjects who were recruited during a 5-year period (2004–2008). A group of 398 newly diagnosed patients with DTC (309 females and 89 males) with a mean age ±s.d. of 46.99±15.45 years was recruited at Vall d'Hebron Hospital (Barcelona) and at Josep Trueta Hospital (Girona). Thyroid cancer patients were classified as papillary cancer (85%) or follicular cancer (15%). The control group consisted of 479 healthy cancer-free volunteers (283 females and 196 males) with a mean age ±s.d. of 45.99±17.26 years. All subjects were Caucasians with Spanish ancestry and from the same geographic area (Table 1).

Individual information was obtained by personal interview, and clinical information of patients was obtained from medical history. All participants in the study gave written informed consent. The study was approved by the ethics committees of all the institutions involved.

### Single nucleotide polymorphisms selection

**Figure 1:**
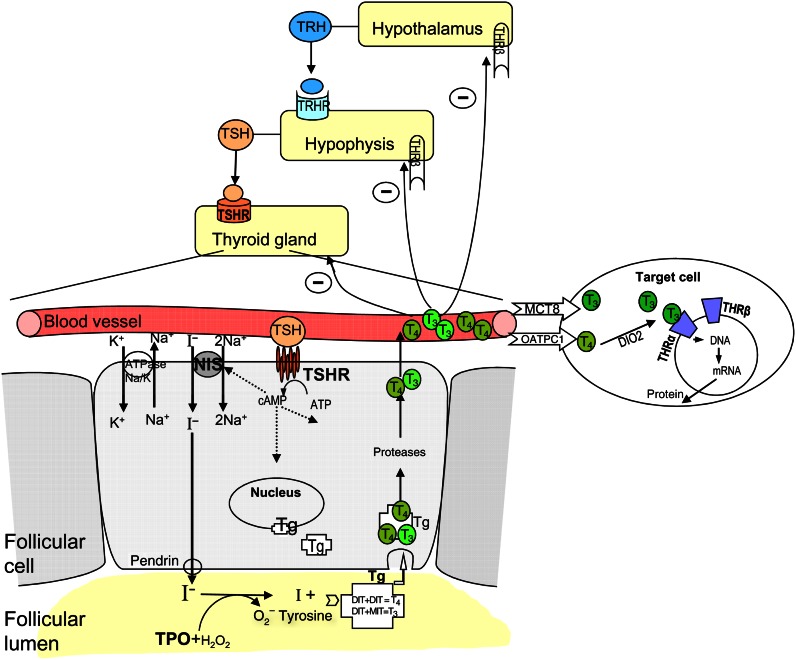
Scheme of the thyroid hormone production and regulation on the hypothalamus–pituitary–thyroid axis.

THRA and THRB and TSHR receptors are critical for the function of the thyroid gland via the pituitary–thyroid axis (see Fig. 1). The selection of the single nucleotide polymorphisms (SNPs) in these genes was based on the information available in the literature and public databases. The selection criteria were based on the minor allele frequency >0.1. A total of six tagSNPs at the *THRA*, *THRB* and *TSHR* genes were selected for the study. More detailed information of the studied SNPs is described in Table 2.

### DNA preparation and genotyping

DNA for both cases and controls was isolated preferentially from peripheral blood mononuclear cells using the standard phenol–chloroform method. For some controls, DNA was obtained from saliva samples using the Oragene DNA Self-Collection kit (DNA Genotek, Ottawa, Ontario, Canada). DNA concentration and purity were measured with a Nanodrop 1000 spectrophotometer (Thermo Scientific, Waltham, MA, USA). The samples were stored at −20 °C until use.

All genotype analyses were performed at the Spanish National Genotyping Centre (CeGen, Santiago de Compostela, Spain) using MassArray Sequenom technology (Genome, San Diego, CA, USA) with the iPLEX strategy. SNP genotyping was performed using a custom-made SNP panel. Genotyping reliability was guaranteed through double genotyping by replicating 10% of samples at random in multiple 96-well plates. In addition, two HapMap reference trios were incorporated in the plates, and the genotype concordance and correct Mendelian inheritance were verified.

### Statistical analysis

The SNPStats software (30), SPSS (PASW statistics v17.0) and GraphPad software were used to perform all the statistical analyses. Comparison of the sex proportion between cases and control groups and the Hardy–Weinberg equilibrium test of the genotype distribution in the control population were examined using the *χ* ^2^ test with a 5% level of significance. Mean ages of cases and controls were compared by the *T*-test. The thyroid cancer risk was assessed using unconditional logistic regression analyses, adjusted for gender and age, to determine the odds ratios (OR) and 95% confidence intervals (CI). Tests for interaction were performed including the cross product of the respective variant alleles. The Haploview software (31) was used to examine the linkage disequilibrium (LD) between adjacent SNPs and to define haplotype block structures. The entire tests were two sided and *P*<0.05 was considered statistically significant.

## Results

The characteristics of the population used in this study are summarized in Table 1. The gender distribution in patients and controls was different (78 and 59% females respectively, *P*<0.0001). Further analysis of the genotype distribution within gender in the control group has showed no statistically significant differences between males and females (data not shown). Thus, in our population, the different sex proportion in patients and control groups did not influence the successive association analysis.

### Association of individual SNPs with risk of DTC

The allele frequencies for the six SNPs studied are shown in Table 2. No significant differences in the allele frequency were observed between cases and controls for any of the SNPs studied, although for the *THRA*-rs939348 a slight difference in cases with respect to controls was observed (0.28 and 0.25 respectively). The Hardy–Weinberg analysis of the control group showed that the genotype distribution was in equilibrium for the six genotyped polymorphisms (*P*=0.07–0.92).

Table 3 shows the genotype distribution of the SNPs studied and the association analysis with DTC, in the total patient group, and in PTC and FTC subgroups. No significant differences in the ORs were observed between controls and patients for either of the three SNPs genotyped in *THRB* or the two genotyped SNPs in *TSHR*.

In the case of the *THRA* gene, for the rs939348 polymorphism, the logistic regression analysis (co-dominant model) showed an increase in the risk of thyroid cancer. The ORs of the homozygous variant (TT) for this SNP were OR=1.71 (95% CI=0.97–3.03, *P*=0.082) for the total of cases, OR=1.59 (95% CI=0.87–2.91, *P*=0.19) for PTC and OR=2.51 (95% CI=1.01–6.24, *P*=0.098) for FTC. These risk estimates suggest that the variant allele (T) could act as a recessive risk factor for DTC. Thus, we next evaluated the risk assessment for this polymorphism according to the recessive model. As shown in Table 3, a statically significant association of the homozygous variant was observed with DTC (OR=1.80, 95% CI=1.03–3.14, *P*=0.037); moreover, the association of the homozygous variant was also significant for the FTC subgroup (OR=2.70, 95% CI=1.12–6.49, *P*=0.037) and marginally significant for PTC (OR=1.66, 95% CI=0.92–3.01, *P*=0.092). The lack of significant association for the papillary type is most likely due to the low number of homozygous individuals for the variant allele in our population.

### Analysis of gene–gene interactions and risk of DTC

Given the functional relationship between *THRA*, *THRB*, *TSHR* and *TRHR*, we decided to evaluate the combined effect of common genetic variants of these genes on thyroid cancer susceptibility. We performed gene–gene interaction analysis on the pairwise SNPs of either *THRA* or *THRB* with *TSHR*, *TG* and *TRHR*. For the interaction analysis, we used the genotype results of this study together with the *TG* and *TRHR* genotype data of our previous association study (12). In both the studies, the same Spanish population was genotyped. No significant interaction for risk of DTC was observed in the pairwise SNPs that included the *THRA*-rs939348 polymorphism (data not shown), although, as indicated before, this polymorphism showed an individual association with thyroid cancer in the study population (see Table 3).

Because two of the three *THRB* SNPs, rs3752874 and rs826377, are in LD (*D*′=0.98, *r* ^2^=0.36), we explored the interaction of *THRB*-rs3752874 and the third *THRB* SNP, rs844107, with *TSHR*, *TG* and *TRHR* genetic variants. In the interaction analysis of two genes, the stratification of the population leads to a reduction of the number of individuals, decreasing the statistical power of the analysis. Therefore, to eliminate such effect for each *THRB* polymorphism (rs3752874 or rs844107), we combined the heterozygous and the homozygous variant individuals as the variant carriers.

Certain combinations between the *THRB* polymorphisms (rs3752874 or rs844107) and polymorphisms in *TG* (rs2076740) or *TRHR* (rs4129682) genes were significantly associated with the risk of DTC. However, neither of these markers, individually, contribute to the DTC risk (see Table 3 and (12)). The rest of the combinations between the *THRB* polymorphisms and *TSHR*-rs8019570, *TSHR*-rs11845164, *TG*-rs180223, *TG*-rs853326 or *TRHR*-rs7823804 showed no statistically significant association with DTC risk (data not shown).

An interaction effect between genetic variants of *THRB* and *TG* was found for thyroid cancer risk. Table 4 illustrates that *THRB*-rs3752874 (exon 7) showed a significant interaction with *TG*-rs2076740 (*P*_interaction_=0.014). The variant allele carriers for *THRB*-rs3752874 (CT+TT) together with the homozygous variant for *TG*-rs2076740 manifested a 0.035-fold decrease in DTC risk (95% CI=0.16–0.79). A combination effect with borderline significance (*P*_interaction_=0.099) was also observed between *THRB*-rs844107 and *TG*-rs2076740.

The interaction between *THRB* and *TRHR* showed that the *THRB*-rs3752874 (exon 7) polymorphism together with *TRHR*-rs4129682 also had a protective effect for DTC risk (*P*_interaction_=0.0024) (see Table 4). The highest protective interaction was found between the *THRB*-rs3752874 variant allele carriers and the *TRHR*-rs4129682 common allele homozygous (CC) that showed a 0.41-fold decrease in DTC risk (95% CI=0.21–0.76).

## Discussion

TRs together with the TSHR have crucial roles in the pituitary–thyroid axis (see Fig. 1). The *TSHR* gene is expressed in the thyroid follicular cells. The *THRA* and *THRB* genes are not only ubiquitous transcription factors but also key regulators of the thyroid function (32, 33). Therefore, we postulated that genetic variants in *THRA*, *THRB* and *TSHR* genes individually or in combination with genetic variants in other thyroid function/regulator genes (i.e. *TG* and *TRHR*) could be important in DTC development. In this study, we provide evidence for the association of *THRA* with DTC. In addition, we show that an interaction exists between *THRB* and *TSHR*, *TG* or *TRHR* that modifies the risk of developing DTC.

In the population studied, the *THRA*-rs939348 in the 5′-noncoding region (intron 2) acted as a possible marker for thyroid cancer susceptibility. According to the co-dominant model, the homozygous variant showed an increased risk of 1.71 (95% CI=0.97–3.03; *P*=0.082). Although the statistical significance with the recessive model was higher than with the co-dominant model (*P*=0.037; see Table 3), these results should be taken with caution, as the number of the homozygous variant was small in our population. Limited information is available about genetic variants in *THRA* related to thyroid cancer. In three earlier reports (21, 22, 23), the dinucleotide CA repeated in the 3′-noncoding region of *THRA* was found to be related to thyroid cancer. Two of these studies were based in a case–control design (22, 23), but the conclusions were limited by the small size of the population used. To some extent, our study, using a population of 877 individuals, confirms the above previous results. In this study, we have examined a *THRA* polymorphism that maps in the 5′-UTR region, far away from the 3′-UTR CA repeat analysed in the previous studies, and our results also indicated a role for the *THRA* gene in thyroid cancer risk. Moreover, Onda *et al*. (21) have reported a correlation of the 3′-UTR CA repeat with *THRA* expression and thyroid tumour aggressiveness. In contrast, no mutations in the *THRA* gene have been described in thyroid tumours (18); thus, the specific function of *THRA* in thyroid cancer remains unclear. More recently, Zhu *et al*. (20) have shown that a mouse model with deleted *Thra* and *Thrb* genes developed FTC, suggesting a tumour suppressor function of these genes in FTC.

Regarding the association of *TSHR* with thyroid cancer, none of the two genetic variants of *TSHR* included in our study (rs8019570 and rs11845164) modified the susceptibility to DTC significantly, suggesting a lack of influence of these polymorphisms on the development of DTC. Indeed, the role of *TSHR* in thyroid cancer is not clear; however, it is a major thyroid autoantigen, and genetic variants in the *TSHR* gene have been described to be associated with the risk of thyroid diseases (29). *TSHR* mutations in DTC are unusual (29), but Xing *et al*. (26) reported epigenetic silencing of *TSHR* in thyroid carcinomas. Two studies investigated *TSHR* polymorphisms related to DTC, although no association was found (27, 28). Altogether, our results confirm previous studies supporting the lack of association between common genetic variants of *TSHR* and DTC risk.

In this study, we found no significant association of any of the three *THRB* SNPs with thyroid cancer when analysed individually. Of these SNPs, rs3752874 and rs826377 map in the ligand binding domain of the *THRB* gene and they showed LD (*D*′=0.98, *r* ^2^=0.36) in our population. The third *THRB* SNP maps in the 3′-UTR region of the gene. Genetic variants in the ligand binding domain of the *THRB* gene have been reported to be associated with serum TSH levels (32, 34), while the 3′-UTR is involved in the silencing of *THRB* by microRNAs in PTC (19). It is also well established that *THRB* is important in thyroid cancer development (33). Recently, the activity of *THRB* as a tumour suppressor has been reported (20, 35), which is correlated with a reduced *THRB* expression found in renal cancer (36, 37, 38). In mice, certain *Thrb* mutations also promoted the development of mammary tumours (39). As a further contribution to all this evidence supporting the role of *THRB* in cancer and particularly in thyroid cancer, the present results represent, to our knowledge, the first study investigating the relationship between *THRB* variants and DTC risk and suggest that individual genetic variants of *THRB* are not related to DTC risk. Nevertheless, it is important to replicate these results in other populations and evaluate other genetic variants of *THRB* to ascertain the implication of individual *THRB* polymorphisms in thyroid cancer susceptibility.

Furthermore, increasing evidence indicates that, together with environmental factors, the interaction of common genetic variants of low penetrance could be major determinants for thyroid cancer susceptibility (3, 40). Thus, we have explored the interaction of the *THRB* gene and other thyroid function-related loci for DTC risk. The reasoning behind these analyses was based on the well-known relevance of *THRB* in DTC, together with the fact that in this study neither of the individually analysed *THRB* SNPs showed association with DTC. We found a gene–gene interaction of *THRB* with *TG* or *TRHR* for thyroid cancer susceptibility in the population studied. The significant *P*_interaction_ values found in this population support the general notion that often no association is detected for individual SNPs, but their effect is manifested in combination with other SNPs. The *THRB* SNP that maps in the 3′-UTR region (rs844107) suggests a combined effect with *TG* for thyroid cancer risk. As the *THRB* 3′-UTR is involved in the silencing of the *THRB* gene (19), we can speculate that genetic variants in the gene encoding the TG protein may produce a small variation in the function of the protein that, in combination with a decrease in the TR, would alter the susceptibility to thyroid cancer. The *TG* polymorphism (exon 33, rs2076740), which has been shown to interact with the 3′-UTR *THRB* polymorphism for DTC risk, maps in a cysteine-repetitive element in *TG* that has been suggested to be involved in intracellular hormone transport (41). This *TG* polymorphism is associated with autoimmune thyroid disease (42, 43) but showed no association with DTC (12). Therefore, another possible explanation for the combined effect of *TG* and *THRB* for thyroid cancer susceptibility could reside in the alteration of the transport of the thyroid hormone together with a decrease in the hormone receptor in the follicular cells of the thyroid gland. On the other hand, the genotyped *THRB* SNP that maps in the hormone binding domain (rs3752874) has shown a combined effect with either the *TG*-rs2076740 or the *TRHR*-rs4129682 for thyroid cancer risk, and the interaction effect was more than multiplicative (*P*_interaction_<0.05 and <0.01 respectively). The combination of *TG* and *THRB* variants could alter the hormone–receptor interaction for DTC risk. Although no functional interaction is expected between THRB and TRHR, we suggest that variations in the function of these proteins could alter the regulation of the thyroid function via the hypothalamic–pituitary–thyroid axis with consequences in the risk for DTC.

In conclusion, this study confirms the association of *THRA* with thyroid cancer in a Spanish population, and it supports the lack of association of *TSHR* with thyroid cancer as previously reported. Furthermore, this is the first study reporting on the analysis of the association of genetic variants in *THRB* with thyroid cancer. The essential role of THRB in thyroid function has been well documented. Here, our results suggest the importance of genetic variants in *THRB* in combination with polymorphisms in other genes of the thyroid function (i.e. *TG* or *TRHR*) in determining thyroid cancer risk. In line with other authors, our study indicates that the role of gene–gene interaction could be crucial in cancer susceptibility.

## Figures and Tables

**Table 1:** General characteristics of the studied population: control and thyroid cancer groups.

	**Control**	**Cases**	**PTC**	**FTC**
Total	**479**	**398**	**339**	**59**
Male, *n* (%)	196 (41)	89 (22)	75 (22)	14 (24)
Female, *n* (%)	283 (59)	309 (78)	264 (78)	45 (76)
Age: mean±s.d.	45.9±17.3	46.9±15.4	46.3±15.1	50.7±17.1
Male	49.9±18.1	47.5±14.3	46.7±14.0	51.9±15.7
Female	43.2±16.1	46.8±15.8^a^	46.2±15.4^a^	50.3±17.6^a^
Mean age at diagnostic		41.7±15.1	40.9±14.6	46.2±16.8^b^

PTC, papillary thyroid cancer; FTC, follicular thyroid cancer.

a *T*-test, *P*<0.05, between control and cases.

b *T*-test, *P*<0.05, between PTC and FTC.

**Table 2:** Information of the studied SNPs and allele frequencies between cases and controls in a Spanish population.

		**Chromosome**				**MAF**		
**Gene**	**SNP ID**	Number	Position^a^	**Nucleotide change**	**Gene polymorphism**	Control *n*=479	Cases *n*=398	***P* value** b
*THRA*	rs939348	17	35485379	C/T	Intron 2	0.25	0.28	0.25
*THRB*	rs3752874	3	24159999	C/T	Phe245Phe	0.16	0.14	0.23
rs826377	3	24143435	T/C	Intron 9	0.18	0.19	0.91
rs844107	3	24138025	A/G	3′-UTR	0.38	0.37	0.60
*TSHR*	rs11845164	14	80601507	C/T	Intron 2	0.14	0.15	0.60
	rs8019570	14	80619348	A/G	Intron 3	0.14	0.15	0.58

*n*, number of subjects; MAF, minor allele frequency; SNP, single nucleotide polymorphisms.

a Position according to NCBI reference sequence.

b Two-sided *χ* ^2^ test for distribution of allele frequencies.

**Table 3:** Genotype frequencies of the selected SNPs and their association with differentiated thyroid cancer in a Spanish population.

**SNP**	**Genotype**	**Control, *n*** (%)	**DTC, *n*** (%)	**Odds ratio** a (95% CI)	***P* value** b	**PTC, *n*** (%)	**Odds ratio** a (95% CI)	***P* value** b	**FTC, *n*** (%)	**Odds ratio** a (95% CI)	***P* value** b
*THRA*											
rs939348	C/C	259 (54.2)	210 (53.2)	1.00		180 (53.6)	1.00		30 (50.9)	1.00	
C/T	196 (41.0)	151 (38.2)	0.89 (0.67–1.18)		130 (38.7)	0.90 (0.67–1.21)		21 (35.6)	0.84 (0.46–1.53)	
T/T	23 (4.8)	34 (8.6)	1.71 (0.97–3.03)	0.082	26 (7.7)	1.59 (0.87–2.91)	0.19	8 (13.6)	2.51 (1.01–6.24)	0.098
C/C+C/T−T/T	23 (4.8)	34 (8.6)	1.80 (1.03–3.14)	**0.037** **c**	26 (7.7)	1.66 (0.92–3.01)	0.092**c**	8 (13.6)	2.70 (1.12–6.49)	**0.037** **c**
*THRB*											
rs3752874	C/C	331 (69.2)	287 (72.7)	1.00		246 (73.0)	1.00		41 (70.7)	1.00	
C/T	138 (28.9)	103 (26.1)	0.85 (0.63–1.16)		86 (25.5)	0.83 (0.60–1.14)		17 (29.3)	1.05 (0.57–1.93)	
T/T	9 (1.9)	5 (1.3)	0.63 (0.21–1.95)	0.45	5 (1.5)	0.73 (0.24–2.25)	0.47	0 (0.0)	0.00 (0.00–NA)	0.39
rs826377	T/T	320 (67.0)	260 (65.8)	1.00		223 (66.4)	1.00		37 (62.7)	1.00	
T/C	141 (29.5)	124 (31.4)	1.06 (0.79–1.43)		103 (30.6)	1.03 (0.75–1.41)		21 (35.6)	1.29 (0.72–2.30)	
C/C	17 (3.6)	11 (2.8)	0.77 (0.35–1.69)	0.72	10 (3.0)	0.83 (0.37–1.87)	0.87	1 (1.7)	0.50 (0.06–3.93)	0.50
rs844107	A/A	178 (38.5)	158 (40.8)	1.00		135 (40.7)	1.00		23 (41.1)	1.00	
A/G	219 (47.4)	176 (45.1)	0.89 (0.66–1.21)		155 (46.7)	0.93 (0.68–1.26)		21 (37.5)	0.79 (0.42–1.49)	
G/G	65 (14.1)	54 (14.1)	0.92 (0.60–1.41)	0.76	42 (12.7)	0.83 (0.52–1.31)	0.71	12 (21.4)	1.47 (0.68–3.14)	0.31
*TSHR*											
rs11845164	T/T	347 (73.0)	287 (72.7)	1.00		242 (71.8)	1.00		45 (77.6)	1.00	
T/C	122 (25.7)	97 (24.6)	0.97 (0.71–1.33)		86 (25.5)	1.02 (0.74–1.42)		11 (19.0)	0.71 (0.35–1.44)	
C/C	6 (1.3)	11 (2.8)	2.22 (0.79–6.24)	0.29	9 (2.7)	2.08 (0.71–6.06)	0.4	2 (3.5)	2.66 (0.50–14.10)	0.32
rs8019570	G/G	352 (73.6)	288 (72.9)	1.00		243 (72.3)	1.00		45 (76.3)	1.00	
G/A	120 (25.1)	97 (24.6)	1.00 (0.73–1.37)		85 (25.3)	1.04 (0.75–1.44)		12 (20.3)	0.80 (0.41–1.58)	
	A/A	6 (1.3)	10 (2.5)	2.08 (0.73–5.96)	0.38	8 (2.4)	1.90 (0.64–5.70)	0.51	2 (3.4)	2.70 (0.51–14.26)	0.42

PTC, papillary thyroid cancer; FTC, follicular thyroid cancer.

a Adjusted for age and gender.

b *P* value corresponding to co-dominant model.

c *P* value corresponding to recessive model. ***P*<0.05**.

**Table 4:** Risk of DTC associated with the combination of rs3752874 and rs844107 of *THRB* and different *TG* and *TRHR* polymorphisms.

	***THRB* rs3752874**				
CC		CT+TT	
	Controls/cases	OR (95% CI)	Controls/cases	OR (95% CI)	***P* for interaction**
*TG*					
rs2076740					0.014
CC	128/106	1.00	44/46	1.17 (0.71–1.93)	
CT	147/123	0.94 (0.65–1.34)	65/43	0.77 (0.48–1.23)	
TT	40/44	1.30 (0.78–2.16)	30/9	0.35 (0.16–0.79)	
*TRHR*					
rs4129682					0.0024
CC	85/83	1.00	44/17	0.41 (0.21–0.76)	
CT	163/154	0.93 (0.63–1.37)	77/63	0.76 (0.48–1.20)	
TT	80/50	0.57 (0.36–0.92)	25/28	1.14 (0.61–2.15)	
	*THRB* rs844107				
	AA		AG+GG		
	Controls/cases	OR (95% CI)	Controls/cases	OR (95% CI)	*P* for interaction
*TG*					
rs2076740					0.099
CC	66/55	1.00	95/95	1.11 (0.69–1.78)	
CT	81/75	0.97 (0.59–1.59)	127/86	0.76 (0.48–1.21)	
TT	18/23	1.58 (0.76–3.29)	51/30	0.65 (0.36–1.17)	
*TRHR*					
rs4129682					0.11
CC	47/51	1.00	76/46	0.58 (0.33–1.01)	
CT	87/81	0.84 (0.50–1.40)	146/132	0.79 (0.49–1.26)	
TT	42/26	0.52 (0.27–1.00)	60/52	0.74 (0.42–1.29)	

OR, odds ratio adjusted for age and gender; CI, confidence interval.
